# Pro-Oxidant/Antioxidant Balance during a Prolonged Exposure to Moderate Altitude in Athletes Exhibiting Exercise-Induced Hypoxemia at Sea-Level

**DOI:** 10.3390/life11030228

**Published:** 2021-03-11

**Authors:** Antoine Raberin, Elie Nader, Jorge Lopez Ayerbe, Gauthier Alfonsi, Patrick Mucci, Chantal L. Rytz, Vincent Pialoux, Fabienne Durand

**Affiliations:** 1 Laboratoire Européen Performance Santé Altitude (LEPSA), EA 4604, Université de Perpignan Via Domitia, 66120 Font Romeu, France; fdurand@univ-perp.fr; 2 Team « Vascular Biology and Red Blood Cell », Univ Lyon, Laboratoire Interuniversitaire de Biologie de la Motricité (LIBM) EA 7424, Université Claude Bernard Lyon 1, 69000 Lyon, France; elie.nader@free.fr (E.N.); gauthier.alfonsi@sciences.ucly.fr (G.A.); 3 Laboratoire d’Excellence du Globule Rouge (Labex GR-Ex), PRES Sorbonne, 75000 Paris, France; 4 Hospital Universitario German Trias i Pujol, 08911 Badalona, Spain; jlopezayerbe.germanstrias@gencat.cat; 5 ULR 7369 - URePSSS - Unité de Recherche Pluridisciplinaire Sport Santé Société, Univ. Lille, Univ. Artois, Univ. Littoral Côte d’Opale, 59000 Lille, France; patrick.mucci@univ-lille.fr; 6 Department of Physiology and Pharmacology, Cumming School of Medicine, University of Calgary, Calgary, AL T2P 2M5, Canada; chantal.rytz@ucalgary.ca; 7 Libin Cardiovascular Institute of Alberta, Cumming School of Medicine, University of Calgary, Calgary, AL T2P 2M5, Canada; 8 Team « Atherosclerosis, Thrombosis and Physical Activity », Laboratoire Interuniversitaire de Biologie de la Motricité (LIBM) EA7424, Univ Lyon, Université Claude Bernard Lyon 1, 69000 Lyon, France; vincent.pialoux@univ-lyon1.fr; 9 Institut Universitaire de France, 75000 Paris, France; 10 IMAGES ESPACE-DEV, UMR228, Université de Perpignan Via Domitia, 66000 Perpignan, France

**Keywords:** arterial desaturation, oxidative stress, hypoxia, acclimatization, aerobic performance, reactive oxygen species

## Abstract

This study examined to what extent athletes exhibiting exercise-induced hypoxemia (EIH) possess an altered redox status at rest, in response to exercise at sea level (SL) and during moderate altitude exposure. EIH was defined as a fall in arterial O_2_ saturation of at least 4% during exercise. Nine endurance athletes with EIH and ten without (NEIH) performed a maximal incremental test under three conditions: SL, one (H1) and five (H2) days after arrival to 2400 m. Gas exchange and peripheral capillary oxygen saturation (SpO_2_) were continuously monitored. Blood was sampled before exercise and after exercise cessation. Advanced oxidation protein products (AOPP), catalase, ferric-reducing antioxidant power, glutathione peroxidase, superoxide dismutase (SOD) and nitric oxide metabolites (NOx) were measured in plasma by spectrophotometry. EIH athletes had higher AOPP and NOx concentrations at pre- and post-exercise stages compared to NEIH at SL, H2 but not at H1. Only the EIH group experienced increased SOD activity between pre- and post-exercise exercise at SL and H2 but not at H1. EIH athletes had exacerbated oxidative stress compared to the NEIH athletes at SL and H2. These differences were blunted at H1. Oxidative stress did not alter the EIH groups’ aerobic performance and could lead to higher minute ventilation at H2. These results suggest that higher oxidative stress response EIH athletes could be involved in improved aerobic muscle functionality and a greater ventilatory acclimatization during prolonged hypoxia.

## Highlights

EIH athletes exhibited exacerbated oxidative stress at rest and after exercise at SL and after five days of moderate altitude exposure. The specific redox status of EIH athletes is blunted during short term hypoxia. Oxidative stress does not alter performance of EIH athletes.

## 1. Introduction

Altitude exposure is known to reduce the arterial O_2_ pressure thereby increasing levels of oxidative stress [1,2]. Oxidative stress corresponds to an imbalance between pro-oxidant and antioxidant levels, in favor of the former, which can be attributed to increased levels of reactive oxygen species (ROS) [3]. Studies that have focused on exposure to moderate altitude (2000-3000 m) have shown increased oxidative stress marker levels and decreased antioxidant levels after acute exposure (1–24 hours) [4,5]. Moreover, it is known that hypoxia-induced oxidative stress can be a function of the hypoxic dose experienced (intensity and duration) [6]. Oxidative stress may play an adaptive role during exposure to altitude since ROS production is involved in the stabilization of hypoxia-inducible factor 1α [7], known to upregulate ventilatory and hematological adaptations to hypoxia [8]. Nevertheless, oxidative stress may also have detrimental effects, as it has been suggested to have a role in hypoxic pulmonary vasoconstriction [9]. Studies report that exposure to intermittent hypoxia during “living high – training low” protocols induce an increase in oxidative stress that are associated with a greater hypoxic ventilatory response [10,11].

Altitude is not the only cause of hypoxemia, as it is well know that some athletes can exhibit a drop in O_2_ arterial saturation (SpO_2_) during exercise at sea-level (SL) that can be attributed to a fall in arterial O_2_ pressure [12]. This phenomenon called exercised-induced hypoxemia (EIH), can occur in about 70% of endurance athletes [13]. Athletes with EIH have been shown to exhibit a drop in SpO_2_ when exercising over 73% VO_2_max [14] and thus are susceptible to recurring hypoxemic episodes. On one hand, it has been demonstrated that hypoxemia is correlated with oxidative stress [4,15], and in this context, it has been hypothesized that EIH may influence systemic redox status and modify the ability of erythrocytes to transfer oxygen to tissues [16]. Some authors also suggest that oxidative stress could be one of the causes of hypoxemia during exercise at sea level since it is known to increase pulmonary endothelial permeability, which may lead to temporary pulmonary edema [17,18], one of the main mechanisms at the origin of EIH [19].

On the other hand, with the ever-increasing participation in outdoor sports such as trail running, and the development of altitude training in endurance sports, many athletes with EIH are exposed to moderate altitude during training camp and/or competitions. In this context, the potentially altered redox status of EIH athletes could lead to differing adaptations to altitude exposure. Besides hypoxia, exercise is also known to increase oxidative stress [20,21] mainly in a dose-dependent manner [22]. As in normoxia, many studies have shown that exercise during hypoxia promotes ROS production [23,24,25]. Moreover, a combined effect of altitude and exercise has been reported in EIH athletes on the arterial desaturation [26,27,28,29], inducing greater levels of hypoxemia when athletes complete exercise at altitude. Thus, EIH athletes could exhibit greater alterations in the redox status during hypoxic exercise. One can hypothesize that the interaction between EIH, exercise and altitude exposure could lead to higher ROS production in athletes exhibiting EIH at SL. In this context, high oxidative stress may be a target to limit deleterious effect of hypoxemia and further nutritional recommendation could be addressed to EIH athletes.

However, to our knowledge, no study has investigated the pro-oxidant/antioxidant balance in EIH athletes during exercise at moderate altitude although these athletes often face hypoxemic episodes. Therefore, in the present study we evaluated blood oxidative stress levels in EIH compared to non-EIH athletes both at rest and in response to maximal exercise during the first days of acclimatization to moderate altitude (2400 m).

## 2. Materials and Methods

### 2.1. Subjects

Nineteen competitive male cyclists or triathletes were included in the study. Volunteers satisfied inclusion criteria if they were between 18 and 40 years old, were non-smokers and did not suffer from any known cardiovascular, metabolic or pulmonary diseases. Participants’ prestudy training regimes included at least 8 hours a week for the previous 5 years. All participants resided at sea level and did not stay above 500 m for more than one day for at least 3 months preceding the commencement of the protocol. Approval for this study was obtained from local ethics committee that conformed to the Declaration of Helsinki (German Trias I Pujol Hospital, Badalona, Spain for sea-level condition and Health Ministry of Andorra for altitude condition). All participants gave their written informed consent prior to participation.

### 2.2. Protocol

Three testing sessions were performed, one at sea level (SL) and two at altitude: the first testing session occurred after one night of exposure to moderate altitude at 2400 m (short term exposure: H1); and the second testing session occurred after five days of exposure to the same altitude (prolonged exposure: H2). Two days after the SL test, athletes traveled to 2400 m (Pas de la casa, Andorra). Between H1 and H2 conditions, participants trained daily and adhered to strict recommendations: duration of training must not exceed 2 hours for cycling and/or 1 hour for running, while maintaining a heart rate (HR) below 70% of maximal HR recorded at H1. Athletes were not allowed to participate in any intense activity for at least 24 hours before the tests. The participants performed each of the tests at the same time of the day to limit the potential effects of circadian rhythm on the outcomes. The design of the study consisted of performing one maximal incremental test on a cycloergometer in each condition. Before and during exercise, gas exchange parameters, cardiovascular parameters and SpO_2_ were monitored and recorded.

### 2.3. Maximal Exercise Test and Gas Exchange Measurements

The exercise began with a 3 min warm-up at 60 Watts (W). After the warm-up was completed, cycling power was increased by 30 W increments every minute until exhaustion was reached. The test was considered to be maximal if at least three of the following four criteria were met: a change in VO_2_ of < 100 mL with increasing power; a heart rate within 10% of the age-predicted maximal value (210-(0.65 × age) ± 10%), a respiratory exchange ratio (RER) above 1.1 or the inability to maintain the required pedaling frequency (70 rpm) despite maximum effort and verbal encouragement. 

Gas exchange parameters (VO_2_, RER (VCO_2_/VO_2_)), minute volume (VE), breathing frequency (Bf) and tidal volume (VT) were measured through use of a breath-by-breath metabolic analyzer (Quark CPET, Cosmed, Rome, Italy). The analyzer was calibrated according to the manufacturer’s instructions, using a 3 L syringe and a calibration gas containing known O_2_ and CO_2_ concentrations (16% and 5%, respectively).

### 2.4. Arterial O_2_ Saturation and Determination of EIH

The occurrence (or absence) of EIH was determined during the SL test. To assess the occurrence of EIH, peripheral capillary oxygen saturation (SpO_2_) was continuously measured at rest and during the tests with a pulse oximeter probe (Nonin Medical Inc., Plymouth, MN, USA) placed on the ear lobe. The PalmSAT® technology used in the Nonin Medical pulse oximeter yields a measurement accuracy of ± 2.1% relative to the gold standard (CO-oximetry analysis of arterial blood samples). This technique has been most recently used in studies focused on EIH [13,30,31] due to its non-invasiveness and validity [32]. The participants’ ear lobes were prewarmed through use of a vasodilating capsaicin cream (Finalgon, Fher, Spain) to avoid poor tissue perfusion and were then cleaned with alcohol. To maintain permanent skin contact during exercise, the probe was held in place by adhesive tape. EIH was defined by a fall in SpO_2_ of at least 4% between rest and maximal exercise for at least three consecutive minutes during the SL test.

### 2.5. Blood Sampling, Oxidative Stress and Antioxidant Assays

A 5 mL blood sample was obtained from the antecubital vein before and within 1 min after each incremental test. Plasma samples were frozen at −80 °C after centrifugation until analysis was performed less than 6 months following the protocol. Advanced oxidation protein products (AOPP), catalase (CAT), ferric-reducing antioxidant power (FRAP), glutathione peroxidase (GPX), superoxide dismutase (SOD) and nitric oxide metabolites (NOx) were assessed using methods as previously described [6].

Briefly, the plasma AOPP was determined by spectrophotometry and was calibrated with a chloramine-T solution that absorbs at 340 nm in the presence of potassium iodide. The absorbance of the reaction was read at 340 nm. AOPP concentrations were expressed as μmol·L^−1^ of chloramine-T equivalents. Nitric oxide metabolites were quantified as the sum of nitrite and nitrate (NOx) concentrations in the plasma. After nitrate reduction by nitrate reductase, the fluorimetric quantification of NOx was based upon the reaction of nitrite with 2,3-diaminonaphthalene and sulfanilamide. FRAP plasma concentrations were measured at a controlled temperature (37 °C) by spectrophotometry. FRAP concentrations were calculated using an aqueous solution of known Fe^2+^ concentration (FeSO_4_ and 7H_2_O_2_) as a standard at a wavelength of 593 nm. CAT activity was measured using hydrogen peroxide (H_2_O_2_) as a substrate and formaldehyde as a standard. CAT activity was determined by the rate of formation of formaldehyde induced by the reaction of methanol and using CAT as an enzyme. Plasma GPX activity was determined as the rate of oxidation of NADPH to NADP+ after addition of glutathione reductase (GR), reduced glutathione (GSH) and NADPH, using H_2_O_2_ as a substrate. Plasma SOD activity was determined by the degree of inhibition of the reaction between superoxide radicals (O_2_**^.^**^_^) produced by a hypoxanthine–xanthine oxidase system and nitroblue tetrazolium.

### 2.6. Statistical Analysis 

All statistical analyses were performed using SPSS v.20 (IBM SPSS Statistics, Chicago, IL, USA). The effect of incremental tests, conditions (SL, H1 and H2) and groups (with or without EIH) were compared by using a repeated measured two-way ANOVA (at rest and at maximal exercise). If applicable, a Bonferroni correction was applied to locate differences. Significance was considered at a level of *p* < 0.05.

## 3. Results

### 3.1. Anthropometric Data

In accordance with our methods, nine athletes exhibited EIH during the VO_2_max test performed at SL. Ten athletes did not exhibit EIH and were therefore considered the without EIH (NEIH) group. According to the Dempsey and Wagner classification [33], four of the EIH subjects presented mild hypoxemia (95% > SpO_2_ > 93%) and the other five showed moderate hypoxemia (93% > SpO_2_ > 88%). Subject characteristics are presented in Table 1 for both groups. EIH athletes were significantly younger than NEIH, however no differences were found between groups with respect to height, body mass, BMI, training volume or training history.

### 3.2. Arterial O_2_ Saturation, Oxygen Consumption and Performance

Resting and maximal SpO_2_, VE and VO_2max_ values are presented in Table 2. EIH athletes had lower resting SpO_2_ compared to NEIH at both H1 and H2. SpO_2max_ was lower and maximal aerobic power (Watt_max_) was higher in EIH compared to NEIH in all conditions. There was no between-group difference regarding VO_2max_.

Resting and maximal SpO_2_ values decreased at H1 and H2 compared to SL within the EIH group, however no differences occurred between H1 and H2. Interestingly, in the NEIH group, whereas resting and maximal SpO_2_ were also lower at H1 and H2 compared to SL, values were higher at H2 compared to H1. In both groups, VO_2_max was lower and resting VE was higher at H1 and H2 compared to SL. Resting VE also increased between H1 and H2 in both groups whereas maximal VE only increased between SL and H2 in the EIH group.

### 3.3. Oxidative Stress Markers

**Figure 1:**
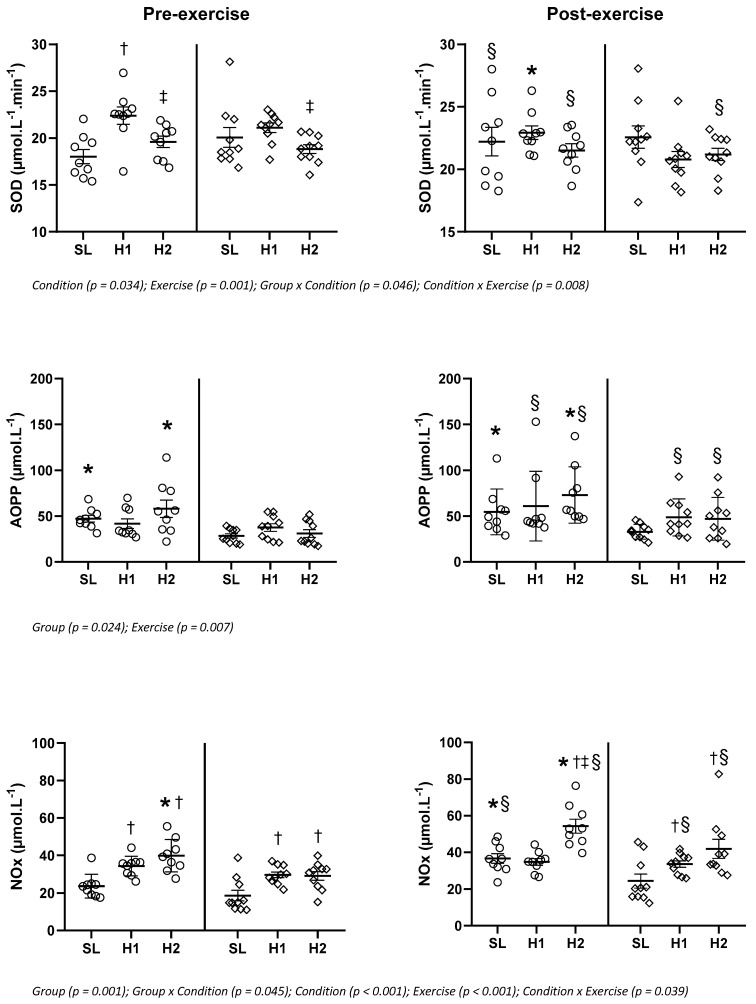
Plasma oxidative stress markers with the main or interaction effect of group exercise-induced hypoxemia (EIH) athletes are circles and NEIH athletes are diamonds. SL: sea level; H1: short-term exposure to altitude; H2: prolonged exposure to altitude; AOPP, advanced oxidation protein product; NOx, Nitric oxide metabolites; SOD, Superoxide dismutase. Values are mean ± SEM. *p* < 0.05 * Different from NEIH, †: different from SL, ‡: different from H1, § different from pre-exercise.

Results regarding oxidative stress markers are reported in Figure 1 when the main effect of group or interaction effect with group was significant or in Table 3 if group or interaction with group were not significant. EIH increased levels of AOPP (*p* = 0.024) and NOx (*p* = 0.005) independently of the condition or exercise. The severity of the arterial O_2_ desaturation during exercise at SL was significantly correlated with AOPP both pre- (r = 0.49, *p* = 0.04) and post-exercise (r = 0.48, *p* = 0.04). EIH athletes exhibited higher levels of AOPP at SL and H2 and of NOx at SL post-exercise and at H2 both pre- and post-exercise than NEIH athletes. Exercise increased NOx in the EIH group at SL (+56%) and H2 (+31%), and at H1 (+13%) and H2 (+34%) in the NEIH group. Resting FRAP concentrations were decreased at H1 compared to SL (−36%) and restored at H2 in the EIH group only. Exercise impacted FRAP at SL (−20%) and H2 (+31%) in the EIH group only. Resting SOD values increased at H1 compared to SL only in the EIH group (+24%) but decreased at H2 compared to H1 in both groups (−13% in EIH and -11% in NEIH groups). Exercise increased SOD at SL (+23%) and H2 (+10%) within the EIH group and only at H2 (+13%) in the NEIH group.

## 4. Discussion

The main finding of this study shows that athletes exhibiting EIH have exacerbated oxidative stress levels at sea level and during prolonged exposure to moderate altitude, however, there was no difference between groups’ oxidative stress parameters in either the exercising or resting conditions after short-term (i.e., one night) altitude exposure.

### 4.1. Oxidative Stress at Sea Level

EIH athletes are subjected to chronic decreases in oxygen saturation [14] that may lead to exacerbated oxidative stress levels [15]. Indeed, even at rest in SL, our results showed a difference amongst redox statuses, where EIH athletes exhibited higher levels of AOPP, a marker of protein oxidation, compared to NEIH athletes. This higher level of AOPP in the EIH group was not accompanied by an increase in any antioxidant markers, suggesting that EIH athletes may overproduce ROS compared to NEIH athletes. This ROS overgeneration may come from activation of the xanthine oxidase pathway, which can occur during the hypoxia/reoxygenation cycle and also through increased ROS production in the mitochondrial complex III, evident during hypoxic conditions [7,34]. Only one study has previously investigated oxidative stress in EIH athletes [16] and reported that EIH did not affect blood redox status at rest or after intense exercise in rowers. Due to its specificity, rowing has a very high prevalence of EIH in elite athletes (100% for Durand et al. (2004) [35]). In the study published by Kyparos et al. (2012), EIH may be due to mechanical ventilatory constraints that are inherent in rowing [36,37] rather than etiological mechanisms associated with oxidative stress, such as stress failure or pulmonary edema [19,38]. Moreover, compared to our study, these EIH rowers were younger and had lower average VO_2_max values (18.3±0.6 vs. 25.5±4.7 years and 60.5±2.2 vs. 69.2±5.6 mL.min^−1^ kg^−1^, respectively), two parameters known to affect oxidative stress levels [39]. Finally, in their study, Kyparos et al. (2012) defined EIH as a drop in SpO_2_ to < 92% during maximal exercise at SL [40] and noted that the normoxemic athletes had a mean saturation of 94.1% ± 0.6%. According to Dempsey and Wagner’s definition (1999), EIH can be categorized from mild (95% > SpO_2_ > 93%) to moderate (93% > SpO_2_ > 88%) and even severe (88% > SpO_2_) hypoxemia. Thus, normoxemic athletes in the Kyparos et al. (2012) study actually exhibited at least mild EIH, perhaps blunting potential differences between the two groups.

It is well know that normoxic exercise can induce ROS overproduction [20]; however our results indicate that EIH athletes express a specific redox status in response to exercise at SL. Indeed, only the EIH group showed increased SOD activity and decreased levels of FRAP post-exercise, and the EIH group had higher levels of both NOx and AOPP compared to the NEIH group post-exercise. The increase in SOD activity may be attributable to an increased production of O_2_**^.^**^_^. Together, these results strongly suggest that oxidative stress in response to exercise is higher in the EIH group. Interestingly, higher oxidative stress could be both a cause [17,18] and a consequence [7,34] of hypoxemia. Although NEIH subjects are significantly older than EIH ones, it is unlikely that this may explain the lower oxidative stress at rest or in response to exercise for NEIH. Indeed, 25 and 33 years old can be considered as the same age group in regards to oxidative stress [41] and higher age rather increases than decreases oxidative stress [42].

### 4.2. Oxidative Stress during Exposure to Moderate Altitude

It has been previously determined that altitude exposure can induce an increase in ROS production and oxidative stress at rest [5,43] and during exercise [21,22]. In our study, independently of the group and exercise, CAT and FRAP levels were decreased and GPX activity was increased at H1 (i.e., short-term altitude exposure) compared to SL and H2. This may reflect an increase in ROS generation since the improvement in GPX activity could be attributed to a higher content of H_2_O_2_. This may also further explain the decrease seen in the antioxidants FRAP and CAT. The discrepancy noted between CAT and GPX activity may be a result of a higher affinity of GPX for H_2_O_2_ compared to that of CAT [44]. The increase in resting SOD activity in the EIH group may be due to O_2_**^.^**^−^ overproduction induced by a lower SpO_2_ experienced by these athletes.

Compared to SL, H1 increased the AOPP response to exercise in both the EIH and NEIH groups, while blunted the SOD activity response to exercise in the EIH group only. These results may indicate an inability of the EIH athletes to face the amount of ROS produced during exercise while experiencing hypoxia since resting SOD activity was already increased to face hypoxia-induced oxidative stress. The imbalance of the redox status results are likely due to an impaired antioxidant status at H1 compared to SL with lower resting levels of FRAP and CAT, and blunted activities of SOD and GPX in response to exercise in both the EIH and NEIH groups.

At H2, resting concentrations of SOD were lower than at H1 and were similar to values seen at SL. This result confirms previous data [4,5,43], which showed that prolonged hypoxic exposure may induce an adaptive antioxidant response to the environment. Indeed, an increase activity of SOD [5,45], GPX [43,45] and catalase [43,45] was reported following acute or prolonged altitude exposure. AOPP, CAT, NOx and SOD increased and GPX activity decreased during exercise in both the EIH and NEIH groups. It could be interpreted that altitude exposure led to a persistent increase in oxidative stress in EIH athletes whereas NEIH athletes require a more prolonged exposure to hypoxia (vs. H1) to exhibit comparable increases in oxidative stress. This higher oxidative stress in response to hypoxia and hypoxic exercise could be, in part, due to the stronger arterial O_2_ desaturation seen in EIH athletes compared to NEIH athletes and also compared to the SL condition. Previous work reported that the changes in plasma oxidative stress levels in response to hypoxic exercise were correlated to arterial O_2_ desaturation [4,15]. Moreover, the higher AOPP and NOx concentrations seen at baseline and after exercise in the EIH group compared to the NEIH group at H2 indicated that the EIH athletes were more susceptible to hypoxia-induced oxidative stress. These results are similar to those at SL, suggesting that five days of exposure to moderate altitude restored the difference in oxidative stress statuses between EIH and NEIH athletes, which did not occur at H1.

### 4.3. Putative Physiological Effects of Oxidative Stress

The impact of excessive ROS production on exercise performance in humans is still a matter of debate. The model proposed by Reid et al. (1993) [46] predicted that when an optimal muscle redox state exists, conditions are ideal for muscle force production, whereas a loss of muscle force can be noted when oxidative stress is increased. In this context, one can hypothesize that EIH athletes exhibited impaired performance due to their higher levels of oxidative stress at sea level and at moderate hypoxia after exercise. However, this increased level of oxidative stress in the EIH group was not concomitant with aerobic muscle dysfunction since for a similar VO_2_max, the EIH group obtained a higher maximal aerobic power than compared to the NEIH group. Although a slightly higher age in NEIH subjects may explain their lower maximal aerobic power, it could be also hypothesized that increased oxidative stress in EIH could be involved in improved muscle contraction–excitation coupling [47] since optimal acute exposure to exogenous ROS generally increases myofibrillar submaximal force.

In our study, we did not find a difference between the two groups (EIH and NEIH) in regards to either the oxidative stress response or a change in ventilation at rest or during exercise at H1. However at H2, the EIH group exhibited higher oxidative stress both pre- and post-exercise and was the only group to show an increase in VE_max_ compared to SL. Thus, it could be hypothesized that EIH may elicit a greater ventilatory acclimatization at H2 due to higher oxidative stress since oxidative stress has been shown to modulate the hypoxic ventilatory response [10,11]. We should however be cautious regarding the direct translation of the present results since plasma oxidative stress, as in our study, may not always reflect redox responses of the cellular compartments that regulate hypoxic ventilatory response.

## 5. Conclusions

Our findings indicated that the EIH athletes exhibited higher levels of oxidative stress at sea level and after prolonged exposure to high altitude both at rest and after maximal exercise. This impact of EIH on the pro-oxidant/antioxidant balance seems blunted by hypoxia during the first day of exposure but restored after five days at 2400 m. In light of our results, this higher oxidative stress response could be involved in improved aerobic muscle functionality and a greater ventilatory acclimatization during prolonged hypoxia. However, with a lower SpO_2_ at rest and during exercise throughout the exposure to hypoxia seen in athletes with EIH, we cannot rule out the putative role of oxidative stress in EIH development and therefore further studies are needed to test this hypothesis.

## Figures and Tables

**Table 1:** Subject characteristics.

	EIH (*n* = 9)	NEIH (*n* = 10)
Age (years)	25.5 ± 1.5 *	33.8 ± 2.3
Height (cm)	181 ± 2	176 ± 2
Body mass (kg)	71.7 ± 2.3	69.6 ± 2.3
Body mass index (m kg^−2^)	21.7 ± 0.6	22.2 ± 0.4
Training volume (h week^−1^)	13.7 ± 1.3	13.5 ± 1.8
Training history (years)	7.3 ± 0.5	8.6 ± 1.9

Values are mean ± SEM. * *p* < 0.05 different from without exercise-induced hypoxemia (NEIH).

**Table 2:** Rest and maximal exercise parameters.

	Group	SL	H1	H2
SpO_2rest_ (%)	EIH	98.7 ± 0.2	94.3 ± 0.4 *†	94.3 ± 0.4 *†
	NEIH	99.1 ± 0.1	96 ± 0.4 †	97.4 ± 0.3 †‡
SpO_2max_ (%)	EIH	93.2 ± 0.3 *	79.7 ± 1.0 *†	81.2 ± 1.1 *†
	NEIH	96.8 ± 0.4	84.7 ± 0.6 †	87.1 ± 0.8 †‡
VE_rest_ (L min^−1^)	EIH	16.0 ± 0.7	18.1 ± 0.7 †	20.0 ± 0.7 †‡
	NEIH	13.8 ± 0.6	15.6 ± 1.4 †	18.1 ± 1.0 †‡
VE_max_ (L min^−1^)	EIH	172 ± 4.9	184 ± 5.5	191 ± 4.9 †
	NEIH	175 ± 7.9	181 ± 6.9	183 ± 7.3
VO_2max_ (mL min^−1^ kg^−1^)	EIH	69.2 ± 1.8	58.4 ± 2.0 †	59.3 ± 1.5 †
	NEIH	65.3 ± 3.0	52.7 ± 1.9 †	55.8 ± 2.2 †
Watt_max_ (Watt)	EIH	465 ± 12 *	416 ± 11 *†	406 ± 10 *†
	NEIH	409 ± 13	366 ± 11 †	362 ± 10 †

SL: sea level; H1: short-term exposure to altitude; H2: prolonged exposure to altitude; SpO_2rest_: peripheral capillary oxygen saturation at rest; SpO_2max_: peripheral capillary oxygen saturation at maximal exercise; VE_rest_: minute ventilation at rest; VE_max_: minute ventilation at maximal exercise; VO_2max_: maximal oxygen uptake, Watt_max_: maximal exercising wattage maintained for at least one minute. Values are mean ± SEM. *p* < 0.05 * different from NEIH; †: different from SL; ‡: different from H1.

**Table 3:** Plasma oxidative stress markers without a main or interaction effect of group.

Condition	Sea-Level		H1		H2	
Exercise	Pre	Post	Pre	Post	Pre	Post
**GPX (µmol L^−1^ min^−1^)**						
EIH	45.4 ± 2.9	46.5 ± 3.9	53 ± 2.6 †	48.5 ± 2.2	52.5 ± 2.4 †	43.9 ± 3.3§
NEIH	45.5 ± 2.7	51.3 ± 3.7	50.8 ± 2.5 †	48.8 ± 2	49.9 ± 2.3 †	44.9 ± 3.2§
*Condition × Exercise (p = 0.040)*						
**Catalase (µmol L^−1^ min^−1^)**						
EIH	95 ± 19	176 ± 37§	37.1 ± 4.0 †	53.9 ± 4.8§ †	50.8 ± 8.4	61.5 ± 9.9§ †
NEIH	110 ± 38	152 ± 40§	28.8 ± 3.8 †	40.9 ± 4.2§ †	19 ± 2.3	45.9 ± 5.2§ †
*Condition (p < 0.001); Exercise (p = 0.009)*						
**FRAP (µmol L^−1^)**						
EIH	532 ± 40	422 ± 38§	340 ± 40 †	355 ± 50	485 ± 60	638 ± 80§‡
NEIH	479 ± 38	436 ± 36	321 ± 38 †	381 ± 48	361 ± 57	450 ± 76
*Condition (p < 0.001); Group × Condition (p = 0.073); Condition × Exercise (p = 0.002)*						

SL: sea level; H1: short-term exposure to altitude; H2: prolonged exposure to altitude; FRAP, ferric reducing antioxidant power; GPX, glutathione peroxidase. Values are mean ± SEM. *p* < 0.05 †: different from SL, ‡: different from H1, § different from pre-exercise.

## Data Availability

Data are available from the corresponding author on reasonable request.
